# Comparative Expression Study of the Endo–G Protein Coupled Receptor (GPCR) Repertoire in Human Glioblastoma Cancer Stem-like Cells, U87-MG Cells and Non Malignant Cells of Neural Origin Unveils New Potential Therapeutic Targets

**DOI:** 10.1371/journal.pone.0091519

**Published:** 2014-03-24

**Authors:** Marie Fève, Jean-Michel Saliou, Maria Zeniou, Sarah Lennon, Christine Carapito, Jihu Dong, Alain Van Dorsselaer, Marie-Pierre Junier, Hervé Chneiweiss, Sarah Cianférani, Jacques Haiech, Marie-Claude Kilhoffer

**Affiliations:** 1 Laboratoire d'Innovation Thérapeutique, UMR7200, Laboratoire d'Excellence Medalis, CNRS, Université de Strasbourg, Faculté de Pharmacie, Illkirch, France; 2 Laboratoire de Spectrométrie de Masse BioOrganique, UMR7178, CNRS, Institut Pluridisciplinaire Hubert Curien, Université de Strasbourg, Strasbourg, France; 3 Neuroscience Paris Seine, UMR8246, Inserm U1130, Institut de Biologie Paris Seine, CNRS, Université Pierre et Marie Curie, Paris, France; Medical School of Hannover, Germany

## Abstract

Glioblastomas (GBMs) are highly aggressive, invasive brain tumors with bad prognosis and unmet medical need. These tumors are heterogeneous being constituted by a variety of cells in different states of differentiation. Among these, cells endowed with stem properties, tumor initiating/propagating properties and particularly resistant to chemo- and radiotherapies are designed as the real culprits for tumor maintenance and relapse after treatment. These cells, termed cancer stem-like cells, have been designed as prominent targets for new and more efficient cancer therapies. G-protein coupled receptors (GPCRs), a family of membrane receptors, play a prominent role in cell signaling, cell communication and crosstalk with the microenvironment. Their role in cancer has been highlighted but remains largely unexplored. Here, we report a descriptive study of the differential expression of the endo-GPCR repertoire in human glioblastoma cancer stem-like cells (GSCs), U-87 MG cells, human astrocytes and fetal neural stem cells (f-NSCs). The endo-GPCR transcriptome has been studied using Taqman Low Density Arrays. Of the 356 GPCRs investigated, 138 were retained for comparative studies between the different cell types. At the transcriptomic level, eight GPCRs were specifically expressed/overexpressed in GSCs. Seventeen GPCRs appeared specifically expressed in cells with stem properties (GSCs and f-NSCs). Results of GPCR expression at the protein level using mass spectrometry and proteomic analysis are also presented. The comparative GPCR expression study presented here gives clues for new pathways specifically used by GSCs and unveils novel potential therapeutic targets.

## Introduction

Glioblastomas (World Health Organization (WHO) grade IV astrocytomas) are highly aggressive, angiogenic and infiltrating brain tumors representing more than 50% of all gliomas [1]. Their outcome is poor, most treatments currently in use remaining inefficient on long term survival [2], [3], [4] and less than 5% of patients survive 5 years post diagnosis [1]. Numerous clinical trials, mainly in phase I/II, are ongoing worldwide to find treatments with increased efficacy (http://clinicaltrials.gov/, http://apps.who.int/trialsearch/and for recent review see [5]). Improvement of the standard Stupp protocol [3], [6], association to topoisomerase inhibitors, alkylating agents, tyrosine kinase inhibitors, intercalating agents or antibodies targeting VEGF or the EGF receptor, development of new combinations of chemotherapeutic agents and testing new delivery drug devices have been used in different protocols (for exhaustive review see [5]). However no therapy appears as the panacea. Seeking for new molecules acting on targets different from those explored so far and improvement of multi-targeted protocols are necessary to decrease recurrence and morbidity of this brain cancer that afflicts humans of all ages.

A new paradigm in cancer therapies arose a decade ago with the identification, within tumors, of cells endowed with stem cell properties and able to propagate tumors in immunodeficient mice with high efficacy. Such cells were first identified in hematological cancers [7]. In 2001, Reya postulated that they may be an integral part of most if not all tumors [8]. Cells with stem properties, long term survival, able to self-renew, differentiate into various cells types and lead to tumor formation after serial transplantation of few cells to immuno-deficient mice were found in many solid tumors including glioblastomas (GBM) [9], [10], breast [11], colon cancer [12], [13], melanoma [14], [15] and pancreatic cancer [16]. Recent experiments, using genetic techniques, allowing the tracing of cancer cells within tumors strongly argue in favor of the presence of tumor stem cells within GBM [17], skin [18] and adenoma [19] tumors *in vivo*. These cancer stem cells (CSCs), also termed tumor stem cells, tumor initiating cells, tumor propagating cells or stem-like cancer propagating cells are considered as the cells responsible for tumor maintenance and relapse after treatment. Classical radiotherapies and chemotherapies which often induce tumor regression are thought to act preferentially on the non-stem cancer cells sparing CSCs and thus causing cancer recurrence. In addition, CSCs are thought to be able to enter a quasi-quiescent state compared to the rest of the tumor cells and thus escape treatments often designed to act on highly proliferating cells. CSCs have thus become the cells to target in order to increase cancer therapy efficacy. Numerous reviews are highlighting this point (for the most recent ones see [20], [21], [22], [23], [24], [25], [26]).

In the present study, we focused our attention on G-protein coupled receptors' (GPCRs) expression in cancer cells with stem and tumor propagating properties isolated from human GBM biopsies [27] and called herein glioblastoma stem-like cells (GSCs). The approach relied on the pivotal role played by this large family of membrane receptors in most key physiological functions including cell communication, cell signalling, cell migration, cell proliferation and apoptosis. These seven trans-membrane domain receptors convey information from outside the cell to inside by binding a variety of physiological compounds of wide chemical diversity. Their paramount importance in cell activity places GPCRs among the most appealing pharmacological targets. This has been extensively exploited to develop major drugs of therapeutic interest for a variety of pathologies including cardiovascular diseases, psychological disorders, allergies, so that nowadays 30–50% of current drugs target GPCRs [28], [29], [30], [31]. Recently, GPCRs also gained attention in anti-tumor drug discovery due to the prominent role they can play, directly or indirectly though transactivation of other receptors, in cancer cell pathophysiology including proliferation, migration/metastasis, angiogenesis and metabolism [31], [32], [33], [34], [35], [36]. The SMO antagonist vismodegib has recently been FDA-approved in basal cell carcinoma [37] and several molecules targeting GPCRs are in clinical trials as adjuvants of chemotherapies (namely Bosentan, an Endothelin A/B subtype receptor antagonist for melanoma or BKT140, a CXCR4 antagonist for multiple myeloma). In addition, the possible use in cancer therapy of monoclonal antibodies targeting GPCRs or their ligands has been reviewed in the literature [33], [38].

In order to get a deeper insight into the pathophysiology of GSCs and find new potential therapeutic targets, we undertook a descriptive study of the set of GPCRs expressed in CSCs isolated from patient primary GBMs in comparison with the GPCR expression repertoires of the GBM cell line U87-MG, non-cancerous human fetal neural stem cells (f-NSCs) and primary human astrocytes (HA). This set of cells was chosen as a first step to compare cancerous and non-cancerous neural cells with stem properties or in a more differentiated state. The study was performed both at the transcriptomic and proteomic levels. The expression profile of this family of proteins appears finely regulated despite karyotype alterations and changes in DNA ploidy [27]. The exquisitely tuned GPCR expression changes observed in the different cell types tested (consisting of cancerous and non-cancerous cells, stem and non-stem cells) lead to a molecular signature evidenced by hierarchical clustering. Finally, the study discloses several GPCRs with high expression specificity in GSCs compared to non-cancerous stem cells or the U-87 GBM cell line. In addition, GPCRs related to stemness have been highlighted. Those GPCRs may give a hook to modify the fate of cancer stem-like cells and more differentiated cancer cells.

## Results

### Global analysis of endo-GPCR gene expression in human GSCs, fetal NSCs, astrocytes in primary culture and GBM U-87 MG cells

Expression of GPCRs was studied in five different human cell types comprising TG1 and OB1 GSCs isolated from GBM biopsies of two patients, the GBM cancer cell line U-87 MG, fetal neural stem cells (f-NSCs) and human astrocytes (HA) in primary cultures. Expression studies were performed by RT-QPCR using the GPCR low density microarrays (Applied Biosystems) in the 384-well plate format. These microarrays allow testing 356 different GPCRs, 2 members of the LANCL family (LANCL1 and LANCL2), which are not considered as GPCRs, 14 different housekeeping genes (with 18 s ribosomal RNA present in quadruplicate) and 11 other genes not related to GPCRs. The list of genes present on the microarray is presented in Table S1. To analyze GPCR expression in the different cell types, data were normalized taking RPLP0, one of the most stable housekeeping genes in our experiments, as reference (Figure S1). The use of RPLP0 alone gave results similar to those using geometric averaging of multiple internal control genes [39]. The results were also similar to those obtained after normalization against TBP previously reported as being an adequate housekeeping gene for expression studies in GBM [40]. In the present experiments, the cycle threshold (Ct) value of RPLP0 was 21.1±0.28. RPLP0 is expressed at a ∼6000 fold lower level compared to the ribosomal RNA 18 s. The most highly expressed GPCRs in each cell type are given in Table 1. GPR56 is highly expressed in all tumor cells but also and even at a higher level, in f-NSCs, the non-tumor stem cells of fetal origin. Its expression in astrocytes is reduced (Ct = 31.2; ratio over RPLP0 = 0.0009). For comparison, low levels of GPR56 were also observed in HEK293 cells, another non-stem cell line (Ct = 30.6 (after normalization); ratio over RPLP0 = 0.001) (data not shown).

**Table 1:** GPCRs most highly expressed in the five cell types.

Cell type	GPCR	Ct	Delta Ct (Ct_GPCR_ – Ct_RPLP0_)	GPCR expression compared to RPLP0 (fold)
TG1	LPHN2	24±0.3	2.88±0.58	7.36±1.49
	GPR56	24.2±0.04	3.08±0.32	8.46±1.25
OB1	F2R	24.3±0.01	3.18±0.28	9.06±1.22
	GPR56	24.4±0.01	3.28±0.29	9.71±1.22
	FZD7	24.5±0.03	3.38±0.31	10.41±1.24
	LPHN2	24.6±0.04	3.48±0.32	11.16±1.25
f-NSC	GPR56	22.6±0.08	1.48±0.36	2.79±1.28
	FZD3	24.7±0.18	3.58±0.46	12.38±1.38
	GPRC5B	24.8±0.15	3.68±0.43	13.09±1.35
HA	FZD1	25±0.08	3.88±0.36	14.72±1.28
	FZD7	25±0.1	3.88±0.38	14.72±1.3
U87	BDKRB2	24.5±0.3	3.38±0.58	10.41±1.49
	GPR56	24.9±0.13	3.78±0.41	13.74±1.33

GPCRs with Ct values ≤25 are indicated. TG1 and OB1 correspond to the GBM cancer stem-like cells. f-NSC, HA and U-87 stand for fetal neural stem cells, human astrocytes and the U-87 GBM cell line, respectively. Ct corresponds to GPCR cycle threshold normalized to the Ct of RPLP0 (Ct = 21.12). GPCR fold expression corresponds to the expression of a given GPCR in a given cell type compared to the expression of RPLP0 calculated as: fold = 2∧^(Ct RPLP0 – Ct GPCR)^.

**Figure 1:**
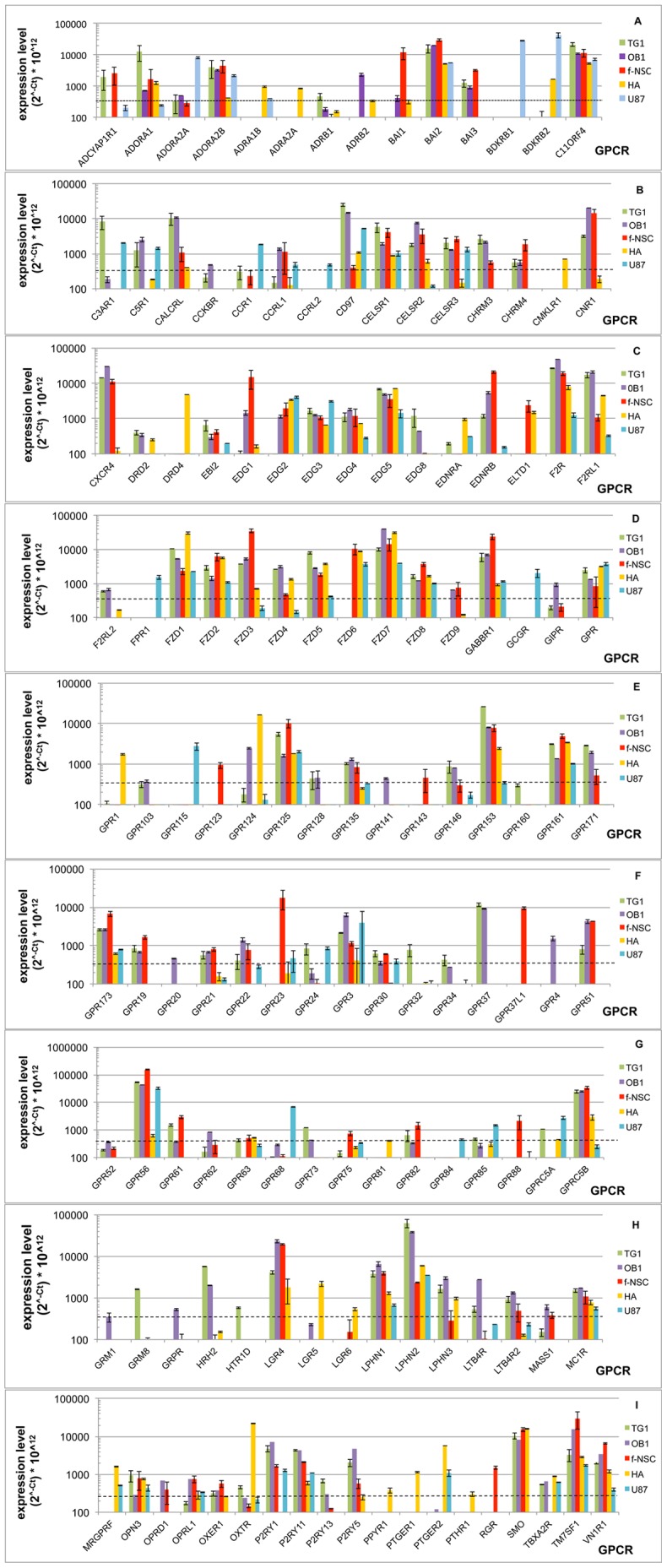
GPCR expression in TG1, OB1, f-NSC, HA and U-87 -MG cells. GPCR expression was determined by RT-QPCR. GPCRs are presented in alphabetical order. Expression levels are related to the cycle threshold value and expressed as 2^∧−Ct^ * 10^∧12^, where Ct corresponds to the cycle threshold of a given GPCR. A Ct of 31.5 corresponds to an expression level of 329 (arbitrary units). Dotted lines indicate the cutoff level (Ct = 31.5). Expression levels are presented on a logarithmic scale. All data were normalized using RPLP0 as housekeeping gene.

Considering the whole set of 356 GPCRs (list in Table S1), we focused our attention on those expressed at Ct values ≤31.5 (i.e. expressed down to 1.33×10^−3^ fold the level of RPLP0) in at least one of the five cell types tested. This stringent condition gives the best signal to noise ratio, although it may leave aside some interesting genes expressed at lower levels. Out of the 356 different GPCRs present on the array, 138 satisfy this criterion (see list and expression values in Table S1). Expression of these 138 GPCRs in the different cell types is represented in Figure 1, where GPCRs are listed, in alphabetical order, as a function of their expression level (expressed as 2∧^−Ct^ * 10∧^12^ (arbitrary units)). Among these 138 GPCRs, 90 genes were expressed above the cutoff level in OB1 cells, 83 genes in TG1 cells, 77 genes in f-NSCs, 61 genes in human astrocytes and 57 genes in the U-87 MG GBM cell line (see expression data in Table S1).

### Hierarchical clustering of GSCs, f-NSCs, human astrocytes and GBM U-87 MG cells using endo-GPCR gene expression

**Figure 2:**
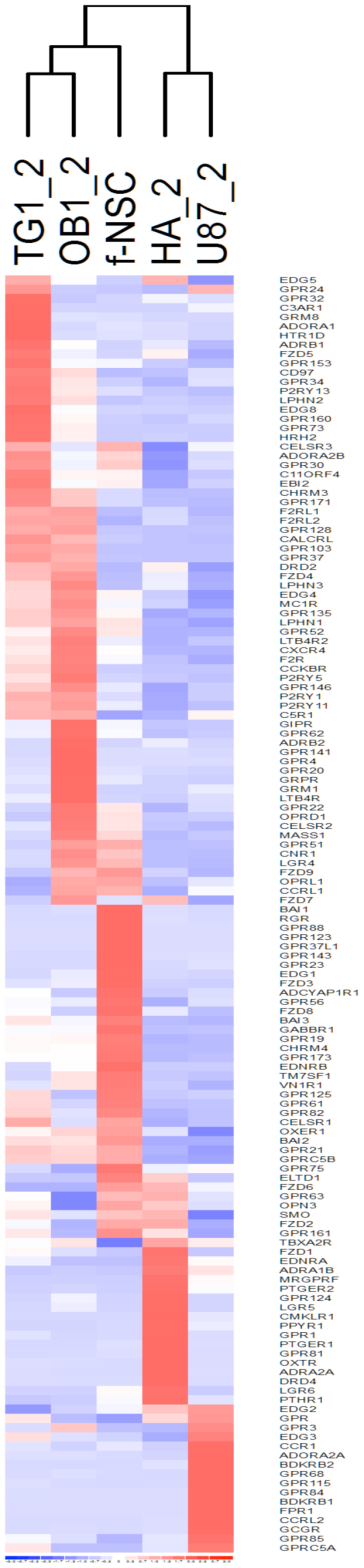
Hierarchical clustering of TG1, OB1, f-NSC, HA and U-87 MG cells based on GPCR expression. The set of 138 GPCRs with Ct values ≤31.5 (or expression levels expressed as 2^∧−Ct^ * 10^∧12^≥329 units) was used for the clustering. For a given cell type, mean expression values (m) from at least two experiments were used for the clustering. Clustering was performed using the DChip software [180].

The 138 GPCRs significantly expressed (with Ct ≤31.5 or expression level >329 units) in at least one of the five cell types studied were used to perform unsupervised hierarchical clustering. Three main clusters could be observed (Figure 2). A first cluster gathers the GSCs TG1 and OB1, the second assembles human astrocytes (HA) and GBM U-87 MG cells and the third cluster corresponds to f-NSCs, which appeared closer to the tumor stem cells than to the astrocytes and GBM U-87 MG cells. The same three clusters are obtained when using all GPCRs present on the microarray without filtering (data not shown). The clustering clearly points to sets of genes specifically expressed or overexpressed either in a given cell type or common to two or more cell types. GPCR expression pattern thus constitutes a signature specific to a given cell type and allows clustering cells as a function of their physiological or pathophysiological states. Of note, the GBM U-87 MG cells cluster apart from the GSCs TG1 and OB1.

Comparative analysis of the differences in GPCR expression among the five cell types, with emphasis on GPCRs specifically expressed in the GBM CSCs will be performed in the following paragraphs.

### Analysis of the core of GPCRs expressed in all of the five cell types

**Figure 3:**
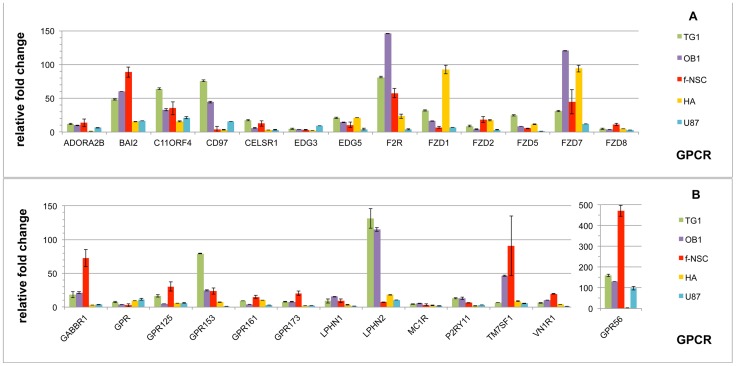
Ensemble of GPCRs expressed in all of the five cell types tested. GPCRs expressed with a cycle threshold (Ct) ≤ to 31.5 in GSCs (TG1 and OB1) human fetal neural stem cells (f-NSCs), human astrocytes (HA) and U-87 MG GBM cells (U-87) are represented. Ct = 31.5 (or expression level expressed as 2^∧−Ct^ * 10^∧12^ = 329 units) was taken as the lowest limit for expression. All data were normalized using RPLP0 as housekeeping gene. Ordinate indicates the fold change in expression of a given GPCR relative to the Ct value of 31.5. Relative fold change is calculated as 2^∧(31.5-Ct_GPCR)^, where Ct_GPCR corresponds to the cycle threshold of a given GPCR in a given cell type. Values correspond to the mean of a least 2 experiments (from 2 to 4). GPCRs are presented in alphabetical order. Note the ordinate scale change for GPR 56.

Among the 138 GPCRs retained in our study, 26 are present at levels above the cutoff value (expression level ≥329 units, i.e. Ct ≤31.5) in the five cell types (Figure 3A and 3B). Receptors of the family of adhesion GPCRs, including BAI2, CD97, CELSR1, GPR125, LPHN1, LPHN2 and GPR56, are the most highly represented (7/26), followed by members of the Frizzled receptor family (5/26) comprising FZD1, FZD2, FZD5, FZD7 and FZD8. The vomeronasal GPCR VN1R1, is also a member of this group of “common GPCRs”. Although expressed in all five cell types, most of the genes present a differential expression level between the five cell types.

As aforementioned, GPR56 is one of the most highly expressed genes in the three cancer cell types tested (TG1, OB1 and U-87 MG). Figure 3 shows that this gene is also highly expressed in f-NSCs. Its expression level in this cell type is respectively 3, 3.6 and 4.8 fold higher compared to that in TG1, OB1 and U-87 MG cells and largely exceeds its expression level in human astrocytes, which is close to the limit fixed in our study. Besides GPR56, the thrombin receptor F2R/PAR1 and to a lesser extent BAI2, GABBR1 and the orphan receptor GPR153 show significantly higher expression in the cells with stem cell properties (f- NSCs, TG1 and OB1), compared to the two other cell types. Only one gene in this group, LPHN2, appears to be overexpressed in the GSCs TG1 and OB1, but not in f-NSCs, nor in the GBM U-87 MG cells or in human astrocytes. Its expression in these two cell types exceeds the mean expression in the three other cell types HA, f-NSC and U-87 MG by 13 fold. On the overall, astrocytes and U-87 MG cells express the “commonly expressed” GPCR genes at the lowest level (with the exception of FZD1 and FZD7 in astrocytes).

### GPCRs with expression specificity in TG1 and OB1 GSCs

**Figure 4:**
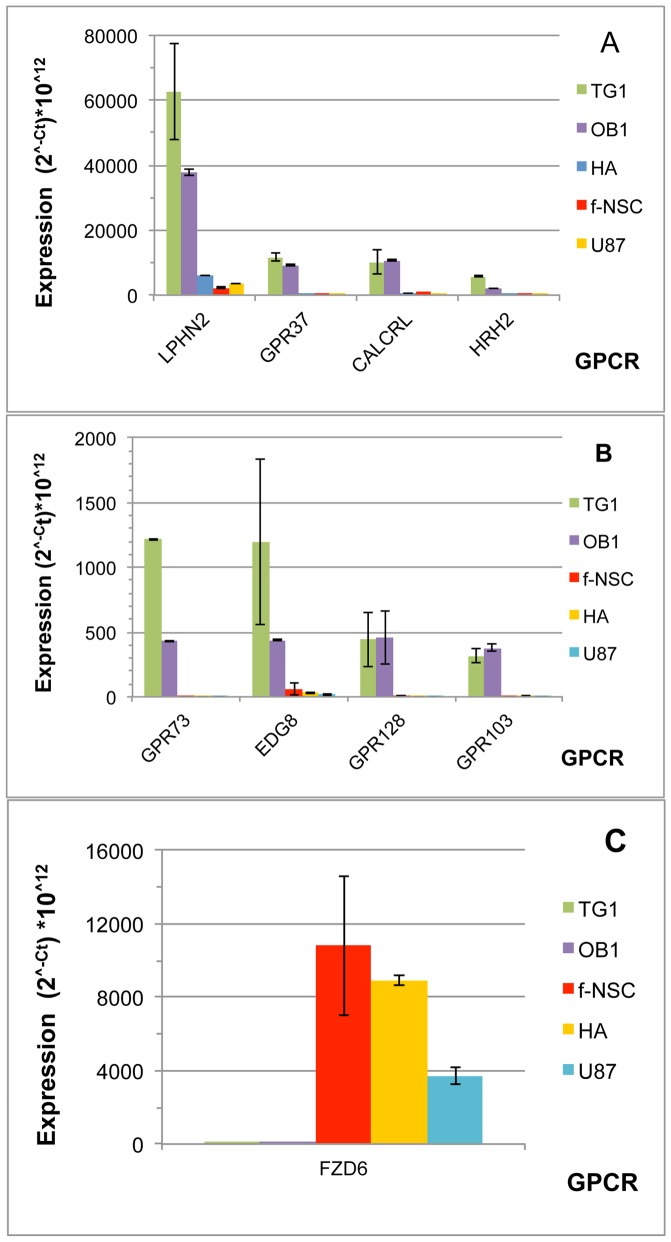
GPCRs preferentially expressed in TG1 and OB1 GSCs. GPCR expression was determined by RT-QPCR. Expression levels are presented as 2∧^−Ct^ * 10∧^12^, where Ct corresponds to the cycle threshold of a given GPCR. A) and B) present GPCRs overexpressed in TG1 and OB1 cells and GPCRs are presented as a function of decreasing expression level in TG1 cells; C) presents the GPCR specifically underexpressed in TG1 and OB1 GSCs. Data correspond to the mean of 4 experiments.

Expression data analyses unveil nine GPCRs with expression specificity in TG1 and OB1 cells (Figure 4). LPHN2, one of these GPCRs, belongs to the group of “commonly expressed” genes (Figure 3 and previous section). However, LPHN2 appears significantly overexpressed in the two GSC cell types compared to the GBM U-87 MG cells or to the non-cancerous cells (Figure 4).

Seven other genes are expressed with specificity in TG1 and OB1 cells. These include CALCRL, GPR37, GPR73, HRH2, GPR103 and GPR128, the levels of the latter two being close to the limit set in our study (Figure 4). The calcitonin receptor-like receptor CALCRL (or CRLR or CLR) is expressed above, but close to the cutoff level in astrocytes and f-NSCs, whereas it is downregulated in U-87 MG cells. Six genes, GPR37, HRH2, GPR73, EDG8, GPR128 and GPR103 are expressed above the cutoff level only in TG1 and OB1 cells. GPR37 is expressed well above the cutoff value in TG1 and OB1 cells and is strongly upregulated in these cells in comparison to f-NSCs, HA and U-87 MG cells. GPR73 expression is increased in both TG1 and OB1 cells, with highest amplitude in TG1 cells. The last three receptors of the group, EDG8, GPR128 and GPR103 are specifically expressed in TG1 and OB1 cells, but at levels close to the cutoff value fixed in the present study. GPR103, although expressed in the lower range level, shows total specificity for TG1 and OB1 cells.

Among the 138 selected GPCRs, only one, FZD6, appears downregulated in TG1 and OB1 cells. FZD6 shows a rather high expression in HA cells and f-NSCs. It is downregulated in U-87 MGcells and absent in the two GSC types. FZD6 belongs to the frizzled family of GPCRs that counts a total of ten members (FZD1-10). Nine FZD genes (FZD1-9) have been analyzed in the present study (Figure 1). FZD10 mRNA could not be detected at a significant level in any of the five cell types tested. Among the nine remaining FZD genes, two may be noticed. FZD3 which shows the highest expression in the cells with stem properties and FZD6, the expression of which is almost completely turned down in the GSCs tested.

### GPCRs showing expression specificity in cells with stem cell properties

**Figure 5:**
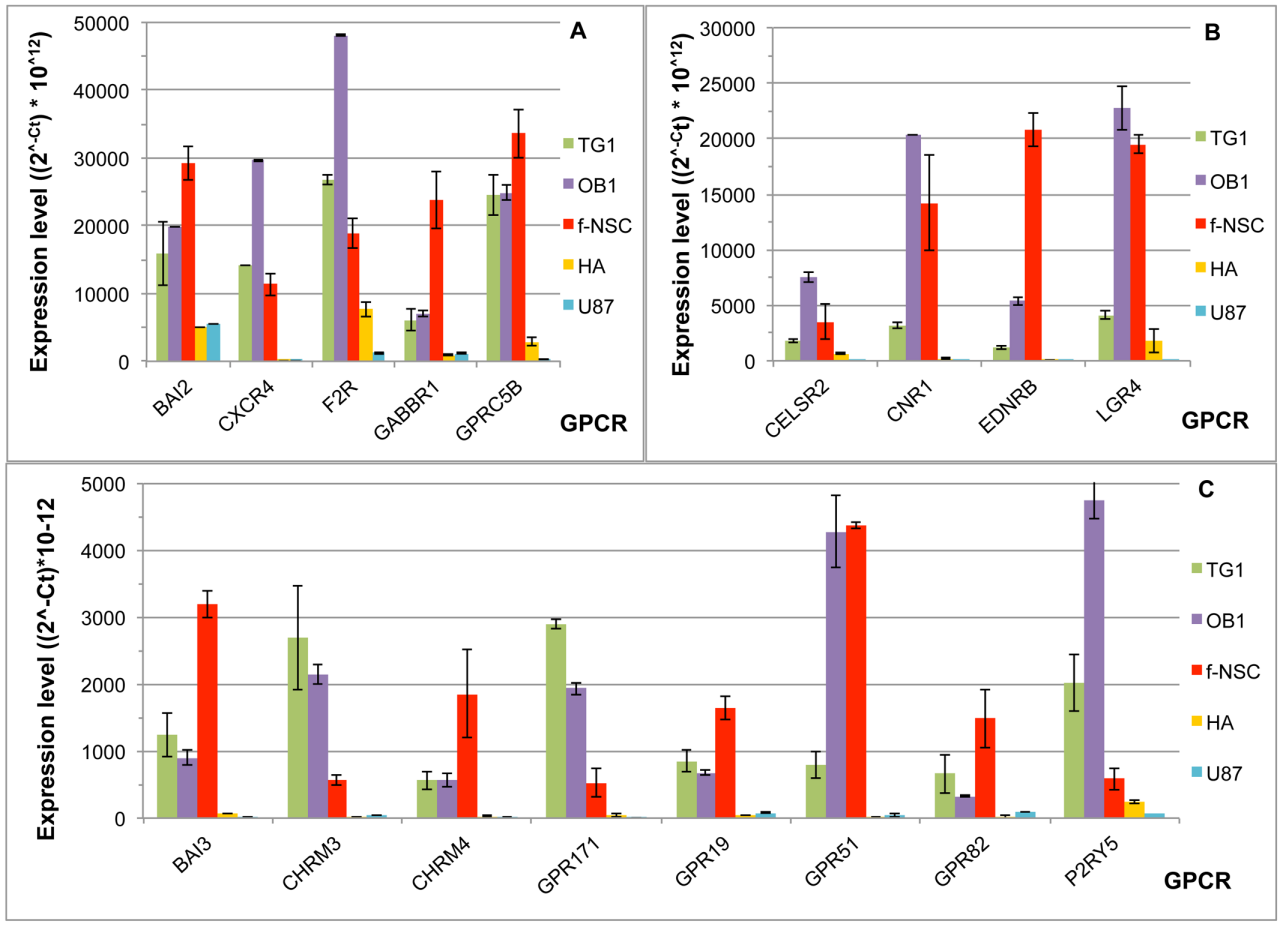
GPCRs overexpressed in the GSCs TG1 and OB1 and the fetal neural stem cells (f-NSCs). The ordinate represents the expression level of the different GPCRs shown as (2^∧−Ct^ * 10^∧12^) where Ct corresponds to the cycle threshold of a given GPCR. Note the difference in scale between A, B and C. HA corresponds to human astrocytes and U-87 to the GBM cell line U-87 MG.

Besides the nine genes with specific changes in TG1 and OB1 cells compared to f-NSCs, astrocytes and U-87 MG cells (Figure 4), other genes show more specific expression in cells with stem properties. Ten genes show expression higher or in the same order of magnitude in f-NSC compared to TG1 and OB1 cells. These include BAI2, CXCR4, F2R, GABBR1 and GPRC5B (Figure 5A) and EDNRB (Figure 5B), BAI3, CHRM4, GPR19, and GPR82 (Figure 5C). Among these genes, CXCR4 appears to be the most specifically expressed in cells with stem properties. Its expression is similar in f-NSCs and TG1 cells, increased in OB1 cells, lower in HA cells and largely downregulated in U-87 MG cells (Figure 5A). Similarly to CXCR4, F2R/PAR1 shows the highest expression in OB1cells. F2R belongs to the Proteinase activated receptors (PAR) also called Thrombin receptors or Coagulation factor II receptors. Two out of the four PARs, namely FR2 (PAR-1) and F2RL1 (PAR-2) are significantly expressed in four (PAR-2) and all (PAR-1) of the five cell types tested, respectively (Figure 1C). Both F2R/PAR-1 and F2RL1/PAR-2 show overexpression in TG1 and OB1 cells, but F2R/PAR-1 has also increased expression in f-NSCs, whereas F2RL1 (PAR-2) has higher expression in HA cells.

Three genes, CHRM3, GPR171 and P2Y5, although expressed in f-NSCs, show higher expression in TG1 and OB1 cells (Figure 5C).

Three more genes LGR4, CNR1 and GPR51 (GABBR2), although expressed in TG1 cells, appear more specific to f-NSCs and OB1 cells (Figure 5B and C).

Finally, CELSR2 expression is also upregulated in these cells, with the highest expression in OB1 cells (Figure 5B).

### GPCRs with higher expression in the cancer cells TG1, OB1 and U-87 MG compared to HA and f-NSCs

We previously showed that GPR56 was overexpressed in TG1, OB1, U-87 MG and f-NSC cells (Figure 2). Further analysis of the GPCR transcriptome indicates that the expression of one GPCR, CD97, appears more specifically related to the cancer phenotype. Among these cancer cells, CD97 expression appears upregulated in cancer cells with stem cell properties compared to U-87 MG cells.

### GPCRs characteristic of a given cell type

**Figure 6:**
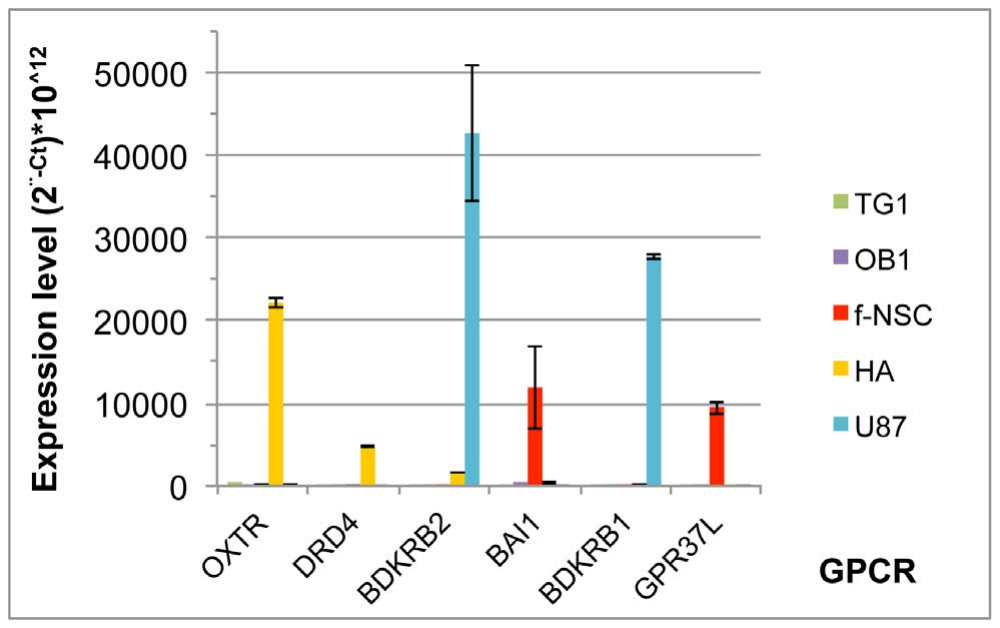
GPCRs specifically expressed in one of the five cell types. Expression of the given GPCR is compared between TG1 and OB1 GSCs, fetal neural stem cells (f-NSCs), human astrocytes (HA) and the GBM cell line U-87 MG. The ordinate represents the expression level of the different GPCRs shown as (2^∧−Ct^ * 10^∧12^) where Ct corresponds to the cycle threshold of a given GPCR. Note the difference in scale between A) and B). TG1 corresponds to GSCs, HA to human astrocytes and U-87 to the GBM cell line U-87 MG.

Analysis of Figure 1 also points to GPCRs that appear highly expressed in only one of the five cell types (Figure 6). Thus, HA cells are characterized by the specific expression of OXTR and DRD4, f-NSCs by BAI1, GPR37L1 and U-87 MG cells by BDKRB1 and to some extent BDKRB2 (although this GPCR is also expressed at low levels in HA cells).

### Proteomic analysis of GPCR expression in human GBM CSCs, f-NSCs, HA and GBM U-87 MG cells

One step further in our investigation was to study GPCR expression at the protein level. Antibodies are often used as tools to investigate protein expression and localization. However the use of antibodies is cumbersome if a great number of proteins have to be analyzed. Moreover, the use of antibodies and especially those targeting GPCRs is often hampered by their lack of specificity. This has been reported for several receptors [41] including α1 adrenergic [42], dopamine [43], beta adrenergic [44], muscarinic receptors [45] and histamine H4 receptors [46]. To overcome the tedious steps needed for generation and validation of reliable antibodies, other approaches are needed. In recent years, high throughput protein profiling, even in complex biological systems, has gained considerable interest thanks to the development of mass spectrometry techniques (MS) coupled to liquid chromatography and bioinformatics.

Thus, in parallel to transcriptomic analysis, we developed a mass spectrometry-based proteomic approach to investigate the expression of GPCRs at the protein level. This task appeared challenging as GPCRs are membrane proteins mostly expressed at low levels. Therefore, a dedicated membrane proteomic approach based on 1D gel protein separation, followed by reduction, alkylation and enzymatic digestion prior to nanoLC-MS/MS analysis of peptide mixtures was developed (see material and methods). With a 1% false discovery rate (FDR), between 3000 and 5000 unique proteins were identified for each cell type (more precisely 4090 for f-NSCs, 3997 for TG1, 3960 for OB1, 3758 for U-87 MG and 3216 for HA cells). This led to more than 7000 non-redundant protein identifications when the results from the five different cell types were combined. Among those proteins, about 20% were annotated as plasma membrane proteins according to their GO annotation: 1043 for f-NSCs, 976 for OB1, 966 for U-87 MG, 963 for TG1 and 837 for HA cells. A final list of seven GPCRs was obtained, among which five (GPR56, LPHN2, F2R/PAR1, GPRC5B and CD97) belong to the fifteen most expressed transcripts (expression level >20000 units) (Table 2). Interestingly, CELSR2 which is expressed at a lower mRNA level (7542 units maximum in OB1 cells) was identified with at least 2 peptides in f-NSCs, TG1 and OB1 cells. A detailed analysis of proteomic results was performed in order to explain why some highly abundant gene transcripts were not detected at the protein level, whereas less abundant ones were. Based on in depth GPCR amino acid sequence analysis, the number of MS detectable peptides was estimated (Table 2). To be best detected in MS, peptides should have: i) molecular weights comprised between 1000 and 3000 Da; ii) extracellular or cytoplasmic regions exposed to trypsin digestion and characterized by lower hydrophobicity than trans-membrane domains and iii) no posttranslational modifications. Considering those criteria, our analyses suggest that, grossly, GPCRs with more than 5 potentially detectable peptides and with gene expression levels over 20000 units were observed in the present proteomic study.

**Table 2:** GPCRs detected by proteomic analysis and comparison to those most highly expressed at the transcriptomic level.

				OB1		TG1		f-NSC		HA		U87	
GPCR Name	Access number	Molecular weight (kDa)	Nb of expected unique peptides	Transcript level (2^−Ct^)*10^12^	Nb of unique peptide (nb of spectrum)	Transcript level (2^−Ct^)*10^12^	Nb of unique peptide (nb of spectrum)	Transcript level (2^−Ct^)*10^12^	Nb of unique peptide (nb of spectrum)	Transcript level (2^−Ct^)*10^12^	Nb of unique peptide (nb of spectrum)	Transcript level (2^−Ct^)*10^12^	Nb of unique peptide (nb of spectrum)
***GPR56***	*Q9Y653*	*78*	*14*	***42820***	***4 (8)***	***52347***	***4 (10)***	***155121***	***8 (37)***	*625*	*nd*	***32369***	***3 (6)***
***LPHN2***	*O95490*	*163*	*50*	***37915***	***9 (15)***	***62684***	***12 (24)***	*2338*	*nd*	*5921*	*nd*	*3495*	*nd*
***F2R***	*P25116*	*44*	*5*	***48045***	***2 (6)***	*26751*	*nd*	*18912*	*nd*	*7701*	*nd*	*1259*	*nd*
**BDKRB2**	P30411	44	0	78	nd	0	nd	1	nd	1655	nd	42664	nd
**FZD7**	O75084	61	3	39613	nd	10182	nd	14788	nd	30943	nd	4011	nd
***GPRC5B***	*Q9NZH0*	*41*	*3*	***24853***	***1 (3)***	***24463***	***1 (4)***	***33650***	***3 (7)***	*2888*	*nd*	*248*	*nd*
**TM7SF1**	O60478	45	3	15303	nd	3322	nd	29968	nd	2855	nd	1743	nd
**CXCR4**	P61073	40	2	29644	nd	14193	nd	11354	nd	120	nd	19	nd
***BAI2***	*O60241*	*173*	*40*	*19770*	*nd*	*15863*	*nd*	***29243***	***1 (1)***	*5050*	*nd*	*5478*	*nd*
**BDKRB1**	P46663	40	2	5	nd	0	nd	9	nd	65	nd	27717	nd
**GPR153**	Q6NV75	65	1	8111	nd	26080	nd	7762	nd	2438	nd	342	nd
***CD97***	*P48960*	*90*	*19*	***14593***	***10 (40)***	***24958***	***9 (31)***	*405*	*nd*	*1079*	*nd*	***5233***	***6 (21)***
**LGR4**	Q8N537	104	8	22726	nd	4135	nd	19526	nd	1804	nd	51	nd
**CNR1**	P21554	53	3	20381	nd	3161	nd	14232	nd	190	nd	1	nd
***CELSR2***	*Q9HCU4*	*317*	*79*	***7543***	***2 (2)***	***1769***	***2 (2)***	***3501***	***8 (12)***	***601***	*nd*	*118*	*nd*

List of the GPCRs with the highest transcription level in at least one of the five cell types (expressed as 2^−Ct^ *10^12^ values >20000) and their identification by proteomic analysis. GPCRs are listed from top to bottom as a function of their mRNA level (the most highly expressed in one or several cell types appears at the top). Number of expected unique peptides corresponds to the unique peptides potentially detectable by MS/MS analysis. Expected peptides were calculated after *in silico* trypsin digestion of a given GPCR taking into account different criteria: i) no miscleavage, ii) molecular weights comprised between 1000 and 3000 Da, iii) presence in extracellular or cytoplasmic regions and iv) no posttranslational modifications. GPCRs identified by proteomic analysis are indicated in italics. OB1 and TG1 correspond to the GSCs. f-NSC, HA and U-87 stand for fetal neural stem cells, human astrocytes and the U-87 MG GBM cell line, respectively. Ct = cycle threshold; nd = not detected).

To obtain semi-quantitative information on proteins identified by mass spectrometry, we performed spectral and peptide counting analysis. For GPR56, one of the best identified GPCRs by proteomic analysis, spectral counting led to an expression pattern similar to that observed at the transcript level, i.e. expression of GPR56 being higher in f-NSCs than in TG1, OB1 and U-87 MG cells and not detected in HA (Table 2). LPHN2 which shows high transcript levels specifically in OB1 and TG1 cells, exhibits similar results at the protein level (LPHN2 is detected with a high number of peptides and spectra in both TG1 and OB1 cells and is not detected in f-NSCs, U-87 MG cells or in astrocytes) (Table 2). Interestingly, all the MS/MS selected peptides are located in the extracellular domain of LPHN2 even if 34 peptides could have been expected from the cytoplasmic domain. This observation could be consistent with cytoplasmic domain processing which eliminates part of the C-terminal domain of the receptor. CD97 is another GPCR for which proteomics follows the trend of transcriptomics. CD97 transcript was found only in the cancer cells with overexpression in GSCs. At the protein level CD97 appears highly overexpressed in TG1 (9 peptides), OB1 (10 peptides) and U-87 cells (6 peptides) while it was not identified in f-NSCs and HA cells (Table 2). Also, CELSR2 which showed a transcript level in OB1>f-NSC>TG1>HA was detected with the same expression hierarchy at the proteomic level with OB1>f-NSC>TG1>HA (Table 2). F2R (PAR-1), GPRC5B and BAI2 belong to the group of GPCRs overexpressed at the mRNA level in cells with stem properties. With only five potentially detectable peptides, F2R was identified by proteomic analysis only in OB1 cells that present the highest mRNA level for this GPCR (Table 2). Despite a lower level of transcript and few peptides detectable by MS/MS, GPCR5B was identified in f-NSCs, OB1 and TG1 cells in agreement with the RT-QPCR analysis (Table 2). Surprisingly, BAI2, a 173 kDa protein for which 40 unique peptides could be expected by MS analysis and with a mRNA level similar to that of CD97 (Table 2), was identified by a single peptide in f-NSCs (the cell type which showed the highest transcript level) (Table 2).

## Discussion

This paper presents the first exhaustive study of GPCR expression at both the transcriptomic and proteomic levels in GSCs. Analysis of the difference in GPCR expression between several cell types of neural origin highlights potential functioning specificities. Investigating the GPCR proteome was challenging as most of these membrane receptors are expressed at low levels. Besides the expression level, receptor accessibility to proteolytic enzymes, which can be related to the length of the extra- and intracellular domains and post-transcriptional modifications, must be considered. Seven GPCRs were detected by MS - based proteomics. For all GPCRs, but BAI2, detection by mass spectrometry correlates with their transcript level and number of potential proteolytic peptides. These results thus demonstrate that MS - based proteomic approaches nowadays allow both identification of proteins in complex mixtures of thousands of proteins and quantification with good confidence. It also reinforces transcriptomic data by showing (for the receptors that could be detected) a good correlation between levels evaluated by RT Q-PCR and mass spectrometry, i.e. the highest protein levels being observed for the highest mRNA levels. Due to the lack of selectivity of most commercial GPCR - directed antibodies, MS appears as an attractive methodology for GPCR expression profiling.

The different GPCRs presented in the result section, with expression specificity in either GBM cancer cells, GBM cancer cells and f-NSCs, GSCs and GSCs/f-NSC) are presented in Table 3 along with their known ligand(s), class, function and chromosome localization. In the discussion we will focus our attention on the most highly expressed genes detected by proteomics and on some GPCRs with specific expression in TG1 and OB1 cells. The relevant GPCRs will be discussed in order to highlight their main known functions and, if documented, their implications in glioma or, more generally, in cancer.

**Table 3:** Summary of the GPCRs expressed with the highest specificity in different subgroups of cells comprising at least the GSCs TG1 and OB1.

Subgroup	Function	Ligand	Class	Chromosomal localisation
**TOU**				
CD97	tumor angogenesis, cell migration, EMT	CD55	B Adhesion/EGF-like familiy	19p13
**NTOU**				
GPR56	brain development; tumor metastasis; cell plasticity	collagen III	B Adhesion	16q13
**TO**				
CALCRL/CRLR	angiogenesis, cell proliferation, antiapoptotic, resistance to hypoxia	adrenomedullin, CGRP	B Calcitonin group	2q32.1
EDG8/S1P5	cell migration/proliferation	lysophosphatidic acid, sphingosine 1-phosphate	A	19p13.2
FZD6	tumor suppressor	Wnt	B Frizzled	8q22.3-q23.2
GPR103	pain. RF-amide Receptor	QRFP family peptides	A	4q27
GPR128	orphan no report	orphan	B Adhesion	3q12.2
GPR37	macroautophagy, glio and neuronal protection	prosaposin/Prosapeptide?	A	7q31
GPR73/PKR1	angiogenesis, cell proliferation, invloved in pain such as GPR103	prokinecitin-1 and 2	A	2p13.1
HRH2	angiogenesis, cell proliferation	histamine	A	5q35.2
LPHN2	EMT in heart development	lectomedin	B Adhesion	1p31.1
**NTO**				
BAI2	unclear (neovascularization, apotosis)	orphan	B Adhesion	1p25.2
BAI3	synapse formation, denditric morphogenesis	C1q-like proteins (C1ql1-4) ?	B Adhesion	6q12
CELSR2	planar cell plority	Cadherin domains - homophilic binding	B Adhesion	11p13.3
CHRM3	Cell proliferation,differentiation, survival. Link with EGFR activation. Implicated in cancer	acetylcholine	A	1q43
CHRM4	Cell proliferation, differentiation, survival. Implicated in cancer	acetylcholine	A	11p11.2
CNR1	energy metabolim; glucose oxidation, ketogenesis, apoptosis, autophagy; stem gene expression	anandamide, 2-arachidonyl-glycerol	A	6q14-q15
CXCR4	cell migration, angiogenesis, invasivness hypoxia controlled, member of the CRG (cooperation regulatory genes).	CXCL12	A	2q22.1
EDNRB	self renewal, migration, proapoptotic	endothelins 1 to 3	A	13q22.3
F2R/PAR-1	Various pathophysiological processes. Participates in EGFR signalling and thus involved in carcinoma.HIF inducible.	proteolytic cleavage of N-terminus	A	5q13.3
GABBR1	cell proliferation	GABA	C Metabotropic GABA(B)R	6p22.1
GABBR2 (GPR51)	cell proliferation	GABA	C Metabotropic GABA(B)R	9q22.1-22.3
GPR19	links with stemness	orphan	A	12p12.3
GPR82	orphan, food intake, not essential	orphan	A	Xp11.4
GPR171		orphan	A	3q25.1
GPRC5B/Retinoic acid-induced gene 2 protein2	Unknown; retinoic acid induced	orphan	C Glutamate metabotropic	16p12.3
LGR4	stem cell proliferation/differentiation; Invasivness metastasis	R-spondin	A	11p14-p13
P2RY5/LPAR2	Sparce information. Involved in cancer	oleoyl-L-alpha-lysophosphatidic acid	A	13q14.2

GPCRs with expression specificity in the different subgroups of cells are listed together with i) their reported function in cancer or their physiological implications (if known), ii) their ligand(s), iii) the GPCR class they belong to and iv) their chromosome localization. T, O, U, N, stands for TG1, OB1, U-87 -MG and f-NSC, respectively. GPCRs in TO thus stands for the receptors specifically expressed in TG1 and OB1 GSCs compared to their expression in the three other cell types; TOU for those expressed in TG1, OB1 and U-87 MG and so on.

### Genes with specific overexpression in GBM cancer cells

One gene, CD97, emerges for its specificity in GBM cancer cells. This GPCR shows significant expression change in the brain cancer cells *vs* non-cancer cells and its expression is highest in cancer cells with stem cell properties (TG1 and OB1). This expression specificity is observed both at the mRNA and protein levels. CD97 is a group II adhesion GPCR, member of the epidermal growth factor-seven transmembrane (EGF-TM7) family. Aberrant expression of this gene has been reported in various cancers, namely thyroid, gastric, pancreatic, and esophageal carcinomas [47] and expression seems to be related to tumor aggressiveness. Recently, CD97 was shown to be upregulated in three different GBM cell lines and to be one of the targets of the transcription factor WT-1 (Wilms tumor protein), the expression of which is also upregulated in gliomas [48]. CD97 was found to be involved in invasiveness and metastasis of prostate cancer, (where it forms heterodimers with the lysophosphatidic acid receptor LPAR1 [49], [50]) and more recently of GBMs [48], [51]. Its overexpression is correlated with decreased survival in patients [51]. The present study clearly shows the expression specificity of this receptor in GBM cancer cells compared to non-cancerous neural cells. Further studies need to be performed to establish which of the possible splice variants [52] are upregulated. So far there are no reports on the physiological role of CD97 in brain. The low expression of this receptor in astrocytes and f-NSCs compared to cancer cells places it as a good candidate for further investigation of its role in cancer in general and GSC cells in particular.

### Genes expressed in GBM cancer cells with and without stem properties and in f-NSCs

GPR56, another adhesion GPCR belonging to class VIII is expressed in GBM cells, but its expression is even higher in f-NSCs. GPR56 is known for its expression in cancer cells, including gliomas [53]. In several tumors (melanoma, prostate cancer) [54], [55], [56], the level of GPR56 expression appears to be inversely correlated to malignancy and metastatic potential. Physiologically, GPR56 plays a key role in brain development, being involved in cortical development and radial migration of neural progenitors (NPC/NSC) from the lateral ventricles to the cortical plate. Collagen has been reported to be its major ligand for this function [57]. GPR56 has been shown to be highly expressed in nestin-positive neural stem/progenitor cells in the ventricular/subventricular zone of human and mouse fetal brains as well as in cultured neurospheres derived from both human and mouse fetal brains [58] [59].. Its expression is downregulated during differentiation [59].

The high expression of GPR56 in f-NSCs and its low expression in astrocytes are in agreement with the pre-cited studies performed on neural stem/progenitor cells. However the present study which compares the expression of GPR56 in GBM cells and f-NSCs shows for the first time that GPR56 can be expressed at the same and even higher level in normal (non-cancerous) stem cells than in cancer cells.

### GPCRs showing expression specificity in cells with stem cells properties TG1, OB1 and f-NSC cells

At the transcriptomic level, 17 GPCRs show specific expression in cells with stem properties (Figure 5A–C; Table 3). Four of them, BAI2, F2R, GPCR5B and CELSR2 are detected at the protein level. Information relative to the role of these receptors in stem cells is limited and their role in CSCs remains to be explored.

BAI-2 is a member of the brain angiogenesis inhibitor family (BAIs) which belongs to group VII of adhesion GPCRs and counts 3 members, BAI1 to 3, located on chromosomes 8, 1 and 6 respectively. BAIs are mainly expressed in brain. This group exhibits an N-terminal CUB (for complement C1r/C1s, Uegf, Bmp1) domain, 3 to 5 thrombospondin type 1 repeats (TSR) discovered to regulate the anti-angiogenic activity of thrombospondin-1, one hormone-binding domain, the evolutionary conserved GPCR proteolytic site (GPS) and multiple glycosylation sites. The three receptors are involved in neo-vascularization and have been reported to be involved in apoptosis and regulation of tumor progression (for review see [60]). The antiangiogenic activity of BAI1 in normal and pathological states is well documented [61], [62], [63]. It is triggered by vasculostatin-40, a 40 kDa fragment induced by matrix metalloprotease cleavage [63]. Tumor-suppressor activity has thus been attributed to BAI1 and its use in cancer gene therapy has been proposed [62]. The angiostatic effect of BAI2 (and BAI3) is less documented. Activation through N-terminal cleavage has been reported for BAI2 [64]. Concerning a role of this receptor family in high grade gliomas, data are scarce and concern global levels of expression measured on tissue samples composed of a mixture of cells [65]. The present study which gives a comparative overview of the transcription expression pattern in five different cell types shows that f-NSCs express BAI1-3 at the highest level (Figure 1). BAI2-3 are overexpressed in cells with stem properties compared to more differentiated cells. Such observations have not yet been reported in the literature. Only the lack of expression of BAI1 in various GBM cell lines, including the U-87 MG cells has been documented [61], which is in agreement with the results of the present study (Figure 1). The different members of the family and namely BAI2 and BAI3 may be involved in functions different from neovascularization, related to stemness maintenance or regulation of apoptosis. Further experiments are necessary to uncover the role of these receptors in GBM and in cancer, more generally. Of the three BAIs, the transcription level of BAI2 is highest. Despite its high molecular weight and high level of mRNA in f-NSCs, TG1 and OB1 cells, BAI2 is only scarcely detected (one peptide) at the proteomic level in f-NSCs (Table 2). This raises further questions concerning the expression of the gene at the protein level or receptor processing in the stem-like cancer cells.

F2R is a second GPCR detected by mass spectrometry with expression specificity in cells with stem properties. F2R belongs to the Proteinase activated receptors (PAR) also called Thrombin receptors or Coagulation factor II receptors which count four members. Two PAR receptors, namely FR2 (PAR-1) and F2RL1 (PAR-2) are significantly expressed in, respectively, four (PAR-2) and all (PAR-1) of the five cell types tested (Figure 1). Both F2R/PAR-1 and F2RL1/PAR2 show overexpression in TG1 and OB1 cells. F2R, but not F2RL1 is also overexpressed in f-NSCs. PAR receptors have plural effects on neural cells. PAR receptors and namely F2R/PAR-1 and F2RL1/PAR-2 are expressed in most tumors (for review see [66], [67]) and are known for their cooperation with EGFR signaling [68]. F2RL1/PAR-2 triggers proliferation, migration, invasion of GBM cells and activation of downstream MAPKs including ERKs after stimulation by the HIF-inducible tissue factor (TF) [69], [70]. F2R/PAR-1, the principal thrombin-activated receptor involved in platelet aggregation and endothelial cell proliferation, has been shown to be overexpressed in various invasive and metastatic tumors and its expression levels directly correlate with the degree of invasiveness of the cancer. A link between angiogenesis and EGFR (via the ligand independent EGFRvIII mutant), tissue factor (TF) and PAR receptors has recently been established in GBM cell lines [71]. In melanoma, cell signaling through PAR releases several factors, such as cytokines, proteases, adhesion molecules and growth factors causing changes in the tumor microenvironment [72]. This has not been shown so far for GBMs. PAR receptors probably play a major role in GBM, and even more generally in cancer, linking the tumor microenvironment, hypoxia and angiogenesis to tumor cell proliferation, migration and tissue invasion. However, PARs also play a major role in normal cell functioning and are expressed, as shown in the present study, in astrocytes and fetal neural stem cells. The overexpression of these receptors in cancer stem-like cells has not been reported so far. F2R is detected by mass spectrometry in OB1 cells that have the highest mRNA level for this receptor (Table 2).

GPRC5B (or RAIG-2), a retinoic acid-inducible orphan GPCR, is a member of the group C of metabotropic glutamate receptor family proteins with short N-termini. It is expressed in human brain [35], [73], [74]. Studies performed on Xenopus suggest a signaling though the non-canonical Wnt/frizzled pathway and a possible cross-talk between the two pathways [75]. This receptor may play an important role in brain maturation and neural circuitry building during brain development. Expression variants, due to alternative splicing have been reported in mouse brain [76]. GPRC5B-v1 is ubiquitously expressed, whereas GPRC5B-v2 is more specific to brain. Nevertheless, besides its implication in behavioral abnormalities, information concerning this receptor family is limited. GPRC5B is almost absent from U-87 MG cells. Its mRNA level is highest in f-NSCs, TG1 and OB1 cells with f-NSC>OB1∼TG1 (Figure 1). Interestingly, mass spectrometry confirms its presence at the protein level with the same relative abundance in the three cell types.

CELSR2 (Cadherin EFG LAG seven-pass G-type receptor 2) is a non-classical cadherin adhesion receptor (group IV of adhesion GPCRs), member of the flamingo subfamily (fmi) characterized by the presence of a seven transmembrane domain [77]. The three members of the family,CELSR1-3, have multiple functions in epithelial and nervous systems and are among the core planar cell polarity (PCP) receptors [78], [79], [80]. A role in maintaining quiescence of long term hematopoietic stem cells has been reported [81]. They play a pivotal role in contact mediated communication. PCP regulation appears to take place through cooperation with the Frizzled receptors and the non-canonical Wnt pathway [81], [82]. Aberrant regulation of these signaling pathways has been suggested to be involved in cancer invasion and metastasis [83], [84]. Although expressed in HA cells, CELSR2 mRNA is overexpressed in cells with stem properties with levels in OB1>f-NSC>TG1. The same order of expression is also found at the protein level. The overexpression of this receptor in CSCs deserves further investigation.

Several other highly expressed GPCRs, namely CXCR4, GABA(B)Rs, LGR4 and CNR1, although not detected by mass spectroscopy, will be discussed.

CXCR4 presents the same relative expression as F2R/PAR1 at the mRNA level (Figure 4A) and similarities in functional properties. CXCR4 is a member of the chemokine receptor family and its endogenous ligand CXCL12 has been implicated in tumor development, including gliomas, facilitating angiogenesis, infiltration and metastasis through modulation of VEGF signaling, PAI-1 (plasminogen activator inhibitor-1) production and membrane type-2 matrix metalloproteinase expression (reviewed in [85], [86]). In agreement with the results of the present study, overexpression of CXCR4 in GBMs has been recognized as an attribute of cells with progenitor/stem properties and CXCL12 promotes specifically the proliferation of these cells compared to more differentiated cancer cells [87], [88]. Of note, the micro-RNA cluster miR 302–367 has recently been shown to inhibit the GSCs'self-renewal and infiltrative properties through CXCR4 repression [89]. Recent studies also show a role of the CXCR4/CXCL12 axis in triggering interaction of cancer cells with microvascular endothelial cells and GBM cancer stem cell transdifferentiation into pericytes [90], [91]. The prominent role of this axis in tumor growth and cancer stem-like cells' interaction with their microenvironment and the perivascular space, designed CXCR4 as a potential target for adjuvant chemotherapies. So far the CXCR4 antagonist BKT140 is in phase I/IIA of a clinical trial for multiple myeloma (http://www.clinicaltrials.gov NCT01010880; for review see [86]). Expression of CXCR4 in neural stem cells has also recently been reported [92]. The present study shows that the expression level of CXCR4 in f-NSCs can reach that observed in GSCs. Using antibodies, CXCR4 was found to be expressed in U-87 GBM cells. Our study indicates low expression of the receptor at the mRNA level in U-87 MG cells (below the cutoff level used in the study (Table S1)). Despite its high transcription level in f-NSCs, TG1 and OB1 cells, the protein could not be detected by mass spectrometry.

GABBR1 and GABBR2 (GPR51) which belong to the metabotropic GABA(B) receptors (class C GPCRs) present a relatively high expression in f-NSCs (Figures 5A and 5C). These two receptors are largely distributed in the central nervous system (CNS) and are particularly present on neurons where they play a role in neurotransmission both at the presynaptic and postsynaptic level. The two receptors function by forming heteromeric complexes of GABA(B) R1/R2 subunits [93], [94], [95], [96], but homodimers [97], higher order oligomers [98] and complexes with other GPCRs have also been reported (e.g. the closely related extracellular calcium sensing receptor CaR [99], the muscarinic receptor M2 [100], as well as with the non GPCR GABA A receptors {Balasubramanian, 2004 #242]. GABA(A)R and GABA(B)R have been shown to exert opposite effects on rodent NSCs. GABA(A)R activation restricts NSC proliferation [101], [102], whereas GABA(B)R activation promotes in vitro neurospheres' development [103]. Besides neurons, GABA(B) R1 and R2 are also present in astrocytes [104] and cells outside the CNS [105], [106], [107]. GABA(B) receptors have been involved in various cancers and the GABA(B) receptor agonist baclofen was shown to reduce cancer development in various animal models [108], [109], [110], [111]. However, GABA signaling remains extremely complex and the multiple possible interaction of GABA(B) subunits with other partners, as well as the regulatory role played by the GABA(B)R2 in receptor trafficking [112]renders assessing a clear cut role of these receptors in cancer difficult. The high expression of the two GABA(B) receptors in f-NSCs may suggest a role in stem cells.

Similarly to GABBR2 (GPR51), LGR4 and CNR1 present an increased mRNA level in f-NSCs and OB1 cells.

LGR4/GPR48, a class A receptor, belongs to the family of leucine-rich repeat (LRR)-containing G protein-coupled receptors (or LGRs) composed of six members. LGR1-3 are hormone receptors. LGR4-6 were deorphanized in 2011 when De Lau and Carmon [113], [114], working on intestinal crypt cells, showed that these receptors bind R-spondin, a molecule secreted by the crypt cells and known as a positive regulator of Wnt signaling. In the study of Carmon, LGR4 and 5 were also shown to interact with the Wnt- binding LRP/Frizzled receptor complex. LGR4-6 have been found in various tissues [115], [116], [117], [118], where they regulate stem cell proliferation and differentiation. Inactivation of the LGR4 coding gene decreases cell proliferation and strongly reduces terminal differentiation of Paneth cells in postnatal mouse intestinal crypts [119]. Physiologically, LGR4 appears to play a key role in organism and tissue development [100], [120], [121], [122]. Expression of LGR4 was also associated with invasiveness and metastatic activity of colon and cervical cancer cells [123], [124]. So far no information is available on LGR4 expression in gliomas. Of note, in our study, among the three R-spondin binding receptors, only LGR4 is expressed at a significant level in TG1 and OB1 cells.

CNR1, which encodes the CB1 cannabinoid receptor (CB1), exhibits an expression profile comparable to LGR4 (Figure 5B). CB1 has a widespread distribution in various brain regions [125] and has two known endogeneous ligands (anandamide and 2-arachidonyl-glycerol). Several genetic studies have been performed to link CNR1 mutations to psychiatric but also metabolic disorders [126], [127], [128]. In rat astrocytes CB1 plays pivotal roles in energy metabolism and activation of this receptor increases the rate of glucose oxidation and ketogenesis, two mechanisms involved in energy supply of the CNS (for review see [125]. CNR1 expression has been reported in various glioma cell lines and receptor agonists trigger apoptosis and autophagy of these cells [129], [130]. However cannabinoids' effect is complex and relies on both ligand concentrations and receptor levels [131]. In glioma stem like cells, CB1 agonists have been reported to modulate the expression of stem genes [132]. In human glioma tissues, increased expression of cannabinoid receptors (CB1 and CB2) has been reported to correlate with higher tumor grades [133]; but this remains controversial [134], [135], [136]. In other tumors, a role of CNR1 in invasion and metastasis has been reported [137], [138] and the endocanabinoid system has been reported as a promising tool to improve the efficacy of steroids in colon cancer [139]. Globally, the cannabinoid system appears to present anti-proliferative, anti-angiogenic and pro-apoptotic properties. Nevertheless, its potential use for treating glioma and may be cancer more generally needs further analysis and is hindered by side effects such as anxiety and depression.

Several less abundant GPCRs of the subgroup of genes expressed in cells with stem properties (Table 3), show specific upregulation in GSCs. These include CHRM3, GPR171 and P2RY5. As for F2R/PAR-1 and CXCR4, CHRM3 activation may synergistically act on the EGFR signaling pathway. Much less is known for P2RY5 and GPR171 and their relation to cancer.

### GPCRs specifically expressed/overexpressed in TG1 and OB1 GSCs

At the transcriptomic level, eight GPCRs appeared specifically upregulated and one downregulated in TG1 and OB1 GSCs (Figure 4 and Table 3) compared to their expression in HA or f-NSCs.

LPHN2 shows the highest expression among the different GPCRs of this group. The specific upregulation of LPHN2 at the mRNA level is corroborated at the protein level and both transcriptomic and proteomic data are in agreement with a level in TG1>OB1. LPHN2 is an adhesion GPCR (group I) belonging to the family of latrophilins composed of three members (LPHN1-3) (for review see [140]). Expression of latrophilin 1 and 3 is mainly found in the central nervous system, whereas LPHN2 is more ubiquitous [140], [141], [142], [143]. LPHN2 shares with the two other members a large N-terminus composed of lectin, olfactomedin and hormone binding domains. The receptors bind α-latrotoxin, the black widow spider venom and this binding triggers strong exocytosis at neuron synapses. LPHN2 however shows lower binding affinities than the LPHN1 and LPHN3 isoforms. Not much is known about the functional role of these receptors in vertebrates. Through their large N-terminus, latrophilins may establish interactions with the extracellular matrix. Such interactions have been reported recently for LPHN1 [144], [145] and LPHN3 [146]. Information on the function of LPHN2 is scarce. It has been reported to play a role in the epithelial mesenchymal transition (EMT) necessary for heart valve development during embryogenesis [143]. In addition, one report indicates a higher expression of the gene in breast tumors [147]. More recently, in a genome wide association study approach aimed at identifying genes involved in the response of tumor cells to paclitaxel, a microtubule cytotoxic used in chemotherapies, a SNP located in the intronic part of the LPHN2 coding gene was identified as being associated with the cellular response to this compound [148]. However there is no further information as to how an intronic SNP in LPHN2 may interfere with the cellular effect of paclitaxel. In the current state of knowledge, one may speculate that the overexpression of LPHN2 in TG1 and OB1 GSCs may be related to EMT, the developmental program often activated during cancer metastasis and regulating the equilibrium between CSCs and the non-stem cancer cells [149]. A recent study using in silico analysis and aiming at finding genes under p53 control, designed LPHN2 as a novel target gene of p53 [150]. LPHN1 and LPHN3 are also upregulated in TG1 and OB1 cells, but both receptors appear less specific than LPHN2 and we did not detect them by mass spectrometry.

Other receptors, namely the parkin-associated endothelin B-like receptor (GPR37 or PAEL-R), the calcitonin receptor-like receptor (CALCRL or CRL), the histamine receptor HRH2, the prokinecitin receptor (GPR73 or PKR1), the endothelial differentiation G-protein coupled receptor 8 (EDG8 or S1P5), GPR128 and GPR103, show specific increase in their mRNA levels in TG1 and OB1 GSCs (Figure 4 and Table 3). However their expression is too low to be detected by mass spectrometry.

Concerning the histamine receptors, only changes in HRH2 could be observed in the present study. HRH1, H3 and H4 were expressed below the threshold level used in the study. HRH2 has a large tissue distribution and is involved in a plethora of physiological functions as reviewed in [151], [152]. As other members of the histamine receptor family [153] it has been reported to play a role in cell proliferation, differentiation energy metabolism and thus to be potentially involved in cancer. HRH2 are found in human mammary and gastric carcinoma cells and several human melanoma cell lines and the HRH2 antagonist cimetidine appears to be endowed with anticancer properties, although its action is not exclusively mediated through HRH2 [154], [155]. Clinical trials using HRH2 antagonists as adjuvant treatment in resected colorectal cancer have also been reported ([156]; for review see [157]), but their real benefits need to be assessed. One additional report points to an antiproliferative activity of histamine mediated through HRH2 in pancreatic cells (PANC-1), with a G0/G1-phase arrest and a modulation of the Bcl-2 family proteins [158]. In our hands, two HRH2 agonists (anthamine and dimaprit) and five antagonists (cimetidine, zolandine, ranitidine, thiotidine and famotidine) tested at 5 and 50 µM for 24 hours did not show a significant effect on TG1 GSCs' proliferation and survival. The specific overexpression of this histamine receptor subtype in TG1 and OB1 GSCs suggests its potential involvement in the cells' pathophysiology that needs further and deeper investigation.

The function of the calcitonin receptor-like receptor **CALCRL** depends on the sub-type of RAMP (receptor-activity modulating protein) to which it is associated. It functions either as a receptor for calcitonin-gene-related peptide (CGRP) when associated to RAMP1 or for adrenomodullin (AM) when associated to RAMP2 or RAMP3 [159]. AM has vasorelaxation properties and its expression is controlled by the hypoxia inducible factor (HIF). Cells overexpressing AM exhibit higher resistance to hypoxia induced apoptosis [160]. AM induces overexpression of anti-apoptotic factors and decreases expression of pro-apoptotic factors [160], [161]. The location of AM close to necrotic zones in tumors suggests a major role of this peptide in the resistance of tumor cells to hypoxia. Numerous data are available on AM and its expression in tumors. However data on its receptor are scarce. A recent work shows that CALCRL and its partners RAMP2 and RAMP3 are present at the mRNA and protein levels in GBM tumors isolated from several patients [162]. Moreover, systemic injections of AM receptor antibodies in animals were shown to inhibit both tumor growth and neo-vascularization of U87 xenografted cells in a dose dependent manner [163]. Nevertheless, information on the distribution, regulation and function of endogenous AM receptors remains poor. Overall, data from the literature suggest a role of AM and its receptor in the survival of tumor cells within the hypoxic regions of solid tumors. AM may induce neovascularization and promote tumor growth. However, in the absence of functional data, it remains difficult to assess the exact relationship between AM, hypoxia, cell proliferation, cell survival and CALCR.

Concerning EDG8/S1P5, the role of the ligand S1P (sphingosine 1-phosphate) in cancer has recently been reviewed [164]. For the other receptors of this subgroup, information relative to their implication in cancer is rare (one report for GPR37 [165]) or lacking.

## Concluding Remarks

GPCRs, which mediate extracellular to intracellular signaling, are key receptors in cell functioning in both physiological and disease conditions. Over the years, a great number of GPCRs have successfully been developed as pharmacological targets in various diseases. However, the complexity of GPCR signaling offered by the possible formation of homo- or hetero oligomers, interactions with a wealth of other proteins, including growth factor receptors [166], [167], [168], [169] and downstream effectors, and its regulatory role in several pathways including the Hippo pathway which coordinates tissue homeostasis [170], unveils bewildering opportunities to target new diseases and fuel drug discovery [31]. Oncology is one of the sectors that may benefit from these new developments as deregulation of GPCR-dependent cell signaling sustains and may even drive tumorigenesis [32], [35], [36]. Exploration of the comparative expression of 356 GPCRs (among which 138 were retained in the present study) in GSCs and in non-cancer neural cells with stem cell properties (human fetal neural stem cells) or without stem cell properties (human astrocytes) and to the established GBM U-87 MG cell line, unveils differences in GPCR expression between the five cell types of neural origin. Several genes, including GPR37, GPR73 (PKR1), GPR103, GPR128, EDG8 (S1P5) and HRH2 appear as the most specifically enriched in TG1 and OB1 cells. LPHN2, which is expressed in the five cell types, is predominantly upregulated in TG1 and OB1 cells. LPHN2 is the only GPCR of the group detected by mass spectrometry. The specific underexpression of FZD6 in TG1 and OB1 cells is noteworthy as FZD6 is the only GPCR showing such a profile. The GPCRs emerging from our analysis are related to metabolic functions, pro-angiogenic activities, cell proliferation, resistance to hypoxia and interactions with the microenvironment (Table 3), an ensemble of characteristics which endow these cancer cells with properties allowing them to be maintained and develop in the specific microenvironment created by the tumor and which may induce death of the normal neural cells. GSCs may originate from differentiated cells that acquired stem properties or from stem/immature progenitor cells that lost their normal regulatory capacities to become cancerous. Whatever the origin, the GSCs may have acquired or retained gene expression found in normal stem/progenitor cells. In this GPCR transcriptome study, several GPCRs show a differential expression pattern in f-NSCs, TG1 and OB1 cells compared to HA or U-87 MG cells. Some GPCRs are already known for their involvement in GBMs. Others have been reported for their involvement in different cancers, but not in brain cancers. To our knowledge, this is the first exhaustive report presenting comparable expression profiles of the whole set of GPCRs in cells from GBM tumors and non-cancerous tissues at the transcriptomic and proteomic levels. Not many GPCRs have so far been reported for their role in GSCs. For those genes that are already documented, our findings mostly correlate with the reported studies obtained using other techniques such as use of agonists and antagonists, or knockout/knockdown experiments. GPCRs specific to GSCs or U-87 MG cells constitute the most appealing ones for cancer therapy. In addition to changes in the level of expression, genomic modifications with mutations that can alter the integrity of the receptor, namely its structure, interaction with partner proteins, affinity for its ligand(s) and downstream signaling have to be considered [171]. Protein altering mutations affecting several GPCRs as well as G protein subunits, have been reported in a study of 441 breast, lung, ovarian, pancreatic, prostate cell types and subtypes [172]. Targeting the finely tuned-GPCR dependent signaling with biased agonists or antagonists or allosteric effectors [173] selected to act on the identified deregulated pathways opens new avenues for more efficient and personalized multi-target oriented chemotherapies. Getting deeper insight into tumor pathophysiology, identifying cancer subtypes and developing system biology approaches to simulate cell intra- and intercellular communication networks will pave the route for disruptive innovation in oncology. The present descriptive presentation of GPCRs exhibiting specific expression changes in GCSs is a first step that calls for future studies at the functional level in order to decipher the precise role of these receptors in sustaining the cancerous phenotype.

## Materials and Methods

### Ethics statement

The biomedical research was conducted according to the declaration of Helsinki, to the French laws and was approved by the institutional review board of Ste Anne Hospital, Paris. Patients have given written informed consent. Isolation and characterization of neural stem cells from human fetal brain at embryonic day 50–55 (Carnegie stage 19–22) were performed under ethical approval from the University Paris-Descartes internal review board using tissue donated with written informed consent after elective termination of pregnancy.

### Cell Cultures

Patients' derived cells were isolated from surgical resections of human primary GBMs provided by the Neurosurgery department of Sainte Anne Hospital in Paris. Patients' tumors were diagnosed as GBMs according to the World Health Organization (WHO) classification system [174] and as malignant glio-neuronal tumor cells according to the Sainte Anne's classification and grading system [175]. Malignant glio-neuronal tumor cells (TG1 and OB1 cells) with stem cell properties were isolated from those biopsies as described in [27]. Their characterization was previously reported [27], [176]. *TP53* and *IDH1* were not mutated in these cells and SHH and NOTCH pathways were active. TG1 and OB1 cells were able to differentiate and to induce tumors when orthotopically grafted (<500 cells) in immune-compromised mice. Cells were expanded in Dulbecco's modified Eagle's: F-12 medium (1∶1) supplemented with N2, G5 (containing bFGF and EGF) and B27 (all from Invitrogen, France) at a density of 3.10^4^ cells/cm^2^. Human fetal neural stem cells (f-NSCs/NSC XXIV) were derived from human fetal brain and characterized as previously described [177]. Cells were cultured in “Neurocult”® NSC Proliferation Medium supplemented with basic fibroblast growth factor (10 ng/ml, bFGF, Peprotech, Rocky Hill, NJ) and with epidermal growth factor (20 ng/ml, EGF, Peprotech, Rocky Hill, NJ). Under the culture conditions used, TG1, OB1 and f-NSC cells grew as neurospheres. For cell expansion, once a week, neurospheres were mechanically dissociated into a single-cell suspension and 90% of their culture medium was renewed. Human Astrocyte primary cells (HA cells, ScienCell Research Laboratories) were expanded in Astrocyte Medium (ScienCell Research Laboratories) according to the manufacturer's instructions. Human brain tumor cells U-87 MG (American Type Culture Collection, ATCC) were expanded in ATCC complete growth medium according to the manufacturer's instructions. For all cell lines, Master Cell Banks were established and Working Cell Banks were derived from these Master Cell Banks for further experiments.

### G-protein-coupled receptor transcriptome

Total RNA was isolated from 10^7^cells of each cell type with TRI Reagent™ (Euromedex, France) according to the manufacturer's instructions and further purified with RNeasy mini kit columns (Qiagen). RNA purity was assessed by absorption spectra analysis using a NanoDrop ND-1000 apparatus (Labtech). Absorption ratios A260/A280 and A260/A230 were comprised between 1.8 and 2.1. RNA integrity and concentration were checked using an Agilent 2100 Bioanalyzer and the RNA 6000 LabChip kit. Only RNA with a RNA Integrity Number (RIN) higher than 8.5 was processed (2100 expert software, Agilent Technologies). 10 µg of total RNA were reverse transcribed to single-stranded cDNA using the High Capacity cDNA Reverse Transcription kit (Applied Biosystems). Real-time PCR analysis was performed using TaqMan® Low Density Arrays (Applied Biosystems). TaqMan® GPCR Gene Expression Arrays correspond to quantitative PCR (Q-PCR) assays spotted onto a 384-well card. cDNA (850 ng) was carefully mixed with 850 µL of TaqMan Universal PCR Master Mix (PE Applied Biosystems). Real-time RT-PCR was performed using an ABI Prism 7900HT apparatus (Applied Biosystems, Foster City, CA). Thermal cycling was carried out for 2 min at 50°C (to activate uracil-DNA glycosylase), 10 min at 94.5°C (activation) and 40 cycles of 30 s at 97°C and 60 s at 59.7°C. Each reaction well contained cDNA derived from about 1.5 ng of total RNA. Experiments were performed in triplicates for f-NSCs and duplicates for the four other cell types.

### Cell membrane preparation, protein extraction and separation

Cells grown as neurospheres were dissociated, washed in PBS and suspended in buffer A consisting of 10 mM Hepes buffer pH 7.4, 2 mM EDTA and protease inhibitors (complete proteases inhibitors cocktail from Roche). Cells were mechanically lysed by pipetting the solution up and down with a P1000 pipette and pressing the solution through a 22G needle mounted on a syringe several times (∼15) until 90% of the cells were lysed. Cell debris were removed by a 10 min. centrifugation at 1560 g. The supernatant was collected and centrifuged for 90 mn at 20 800 g. Cell ghosts were resuspended in buffer A. After denaturation at 100°C in 5% SDS, 5% βmercaptoethanol, 1 mM EDTA, 10% glycerol, 10 mM Tris pH 8 buffer for 3 min, protein samples were fractionated on 1D SDS-PAGE gradient gel (8–15%). Gels were stained with colloidal Coomassie Blue. 90 slices were cut and stored at −20°C prior to mass spectrometry analysis.

### Mass spectrometry proteomic analysis

*In gel* digestion of gel slices was performed as previously described [178]. The resulting peptide extracts were analysed by C18 reversed phase nanoHPLC on a nanoHPLC-Chip/MS system (Agilent Technologies, Palo Alto, USA) coupled to an ion trap amaZon (Bruker Daltonics, Bremen, Germany) mass spectrometer. Peptide mixtures were loaded on a 40 nl enrichment column and separated on a 75 µm×150 mm column packed with Zorbax 300SB-C18 5 µm material (Agilent Technologies, Palo Alto, USA). Trapping was performed during 3 min, at 3.75 µL/min, with solvent A (98% H_2_O, 2% ACN and 0.1% FA). Elution was performed using two solvents, A (98% H2O, 2% ACN and 0,1% FA) and B (98% ACN, 2% H2O and 0,1% FA) at a flow rate of 300 nL/min, using a 2–24% B gradient over 45 min and a 24–40% B gradient over 16 min, followed by 70% B over 5 min.

For tandem MS experiments, the system was operated with automatic switching between MS and MS/MS modes. The 8 most abundant peptides, preferably doubly and triply charged ions, were selected on each MS spectrum for further isolation and CID fragmentation. Then, selected ions were excluded during 0.6 min. The MS/MS scanning was performed in the ultrascan resolution mode at a scan rate of 32 500 m/z per second. The complete system was fully controlled by Hystar 3.2 (Bruker Daltonics).

### Proteomic Data Analysis

Raw data collected during nanoLC-MS/MS analyses were processed and converted into *.mgf peak list format with DataAnalysis 4.0 (Bruker Daltonics). MS/MS data were interpreted using two search engines: Mascot (version 2.3.02, Matrix Science, London, UK) installed on a local server and OMSSA (Open Mass Spectrometry Search Algorithm version 2.1.9; [179]) interfaced for grid computing on an in-house developed and freely available software suite (https://msda.unistra.fr). Searches were performed against a composite target-decoy UniProtKB-SwissProt human entries (TaxID = 9606, 10 July 2012, 40500 total entries). Searches were performed with a tolerance on mass measurement of 0.2 Da for precursor and 0.3 Da for fragment ions. Carbamidomethylation of cysteine residues, oxidation of methionine residues and propionamidation of cysteine residues were searched as variable modifications. Up to one trypsin missed cleavage was allowed. Scaffold software (version 3_6_5 Proteome Science, Portland, USA) was used for validation and false positive rate estimation of protein identifications. For each sample and each search engine, peptides were filtered out according to the cutoff set for protein hits with 1 or more peptides and a false positive identification rate less of than 1%. Further data manipulations were performed with Microsoft Excel. Semi-quantification was performed by peptide and spectral counting.

## Supporting Information

Figure S1 **Expression of 14 housekeeping genes present on the TaqMan GPCR Array Card (Applied Biosystems).** Expression distribution for each housekeeping gene, in the different experiments performed, is given as box plots. Ordinates represent the cycle threshold (Ct) of the quantitative PCR experiment for each housekeeping gene indicated. The middle line in the boxes corresponds to the median. The end of the wishers corresponds to the lowest and highest values within 1.5 IQR (interquartile range) of the lower and upper quartile. Outliers are represented by stars. Abbreviations: 18 s, 18 s ribosomal RNA; GAPDH, glyceraldehyde-3-phosphate dehydrogenase ; POLR2A, DNA dependent RNA polymerase 2A subunit; ACTB, actin β; PPIA, peptidylpropyl isomerase A; PGK1, phophoglycerate kinase 1; B2M, β2-microglobulin; GUSB, glucuronidase β; HPRT1, hypoxanthine phosphoribosyltransferase 1; TBP, TATA-box binding protein; TFRC, transferrin receptor; HMBS, hydroxymethylbilane synthase; IPO8, importin 8; PHGDH, phosphoglycerate dehydrogenase.
(DOCX)

Table S1 **Gene assays present on the GPCR microarray plates and their expression values in five cell types.** 381 different assays are present on the GPCR microarray, allowing the analysis of the expression of 356 GPCRs, 14 different housekeeping genes (rRNA 18 s is in quadruplicate) and 11 other genes (among which two genes of the LANCL family). Expression data are presented in four groups. Group I corresponds to GPCRs expressed with Ct values ≤31.5 (138 genes) in at least one of the five cell types, group II to GPCRs expressed at Ct>31.5 in all five cell types, group III to the housekeeping genes (14 genes) and group IV to eleven genes not related to housekeeping genes or GPCRs. In each group, genes are listed in alphabetical order. Expression values are indicated as cycle threshold values (Ct) and as 2∧^−Ct^ * 10^∧12^ (arbitrary units). The cutoff level was set at Ct ≤31.5 (i.e. expression level ≥329 units). For GPCRs which did not show amplification, Ct values were arbitrarily set at 40.
(XLSX)
