# Supplementary figures and images for: Comparative Expression Study of the Endo–G Protein Coupled Receptor (GPCR) Repertoire in Human Glioblastoma Cancer Stem-like Cells, U87-MG Cells and Non Malignant Cells of Neural Origin Unveils New Potential Therapeutic Targets
Source: PLoS One. 2014 Mar 24;9(3):e91519. doi: 10.1371/journal.pone.0091519 (PMC3963860; doi:10.1371/journal.pone.0091519)

Supplementary materials

Figure S1


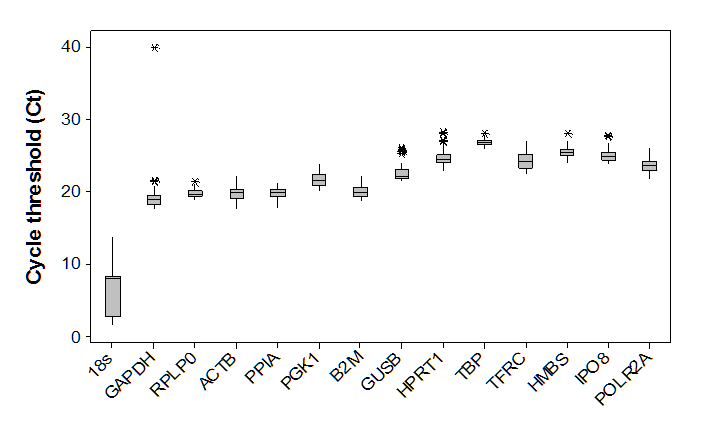

Supplement: Figure S1 — Expression of 14 housekeeping genes present on the TaqMan GPCR Array Card (Applied Biosystems). Expression distribution for each housekeeping gene, in the different experiments performed, is given as box plots. Ordinates represent the cycle threshold (Ct) of the quantitative PCR experiment for each housekeeping gene indicated. The middle line in the boxes corresponds to the median. The end of the wishers corresponds to the lowest and highest values within 1.5 IQR (interquartile range) of the lower and upper quartile. Outliers are represented by stars. Abbreviations: 18 s, 18 s ribosomal RNA; GAPDH, glyceraldehyde-3-phosphate dehydrogenase ; POLR2A, DNA dependent RNA polymerase 2A subunit; ACTB, actin β; PPIA, peptidylpropyl isomerase A; PGK1, phophoglycerate kinase 1; B2M, β2-microglobulin; GUSB, glucuronidase β; HPRT1, hypoxanthine phosphoribosyltransferase 1; TBP, TATA-box binding protein; TFRC, transferrin receptor; HMBS, hydroxymethylbilane synthase; IPO8, importin 8; PHGDH, phosphoglycerate dehydrogenase. (DOCX) [file pone.0091519.s001.docx]
